# The Student Voice Prize: ten years of learning from healthcare students

**DOI:** 10.1186/s13023-026-04237-1

**Published:** 2026-02-24

**Authors:** Lucy McKay, Richard Thompson

**Affiliations:** 1 Medics for Rare Disease, High Wycombe, UK; 2 Beacon for Rare Diseases, Cambridge, UK

Dear editor

Rare Disease Day 2026 will mark a decade of collaboration on The Student Voice Prize, an annual essay competition originally created to increase the engagement of medical students with rare diseases. The collaboration between three parties, Ophanet Journal of Rare Diseases, Medics for Rare Disease, and Beacon for Rare Diseases, has helped to showcase the voice of trainee healthcare professionals around the world. As we celebrate ten years of the collaboration, we have reflected on the competition’s impact and the progress that has been made for the rare community during its lifetime.

## Simple beginnings

The Student Voice Prize was the inspired idea of an intern working for a small rare disease charity, Beacon (then Findacure) to encourage medical students to learn about rare diseases. Whilst the first edition of the competition was very small, with only a handful of entries, both Medics for Rare Disease and Orphanet Journal of Rare Diseases appreciated its unique potential. In subsequent years, the combination of a patient advocacy group, a medical education charity and a medical journal working in partnership, took the competition to the next level. Within three years The Student Voice Prize had expanded significantly with essay entries from eight countries and 23 different universities. The competition had rapidly become a platform for early-stage healthcare professionals to share their experiences and perspectives on rare diseases, in healthcare systems far from optimised for rare care. Entries often reflect on the author’s interactions with people living with rare disease in a clinical setting and occasionally through the personal experience of the author or their family member. From the outset we were keen to capture personal insights from the students so that we could appreciate the views of the next generation of healthcare professionals. Such knowledge would help the rare disease advocacy community to engage with the healthcare workforce in a way that could improve rare care in the future. However we found that students were often impeded from taking part because they had little prior opportunity to learn about rare diseases and to meet impacted patients during their studies.

## Building connections

In 2018 we launched the Patient Group Partnering scheme, allowing student participants to request a direct connection with rare disease patient organisations. Patient Advocacy Groups (PAGs) were encouraged to volunteer for the scheme and be matched with a participant. Students now had the opportunity to hear and reflect on the stories of people with lived experience and PAGs could raise awareness of the unique challenges facing their community. This has not only created long-term bonds but also demonstrated to the patient community that their voices are critical for creating a representative body of literature. One group leader reflected that *“Patient pairing is a brilliant example of a win-win initiative. To have the time to speak with a young medical professional about what really matters in the diagnosis*,* treatment and management of a rare condition is fantastic - especially knowing that they may share this with friends and colleagues*,* as well as through the SVP essay. I’ve taken part for the past 4 years and it has become a mini highlight. I’ve been so impressed with the dedication of the students I’ve talked to.”*.

In 2023 this annual essay competition exceeded 100 entries, representing 18 countries, 30 universities, and the team paired more than 90 entrants with PAGs to support both their learning and writing. This growth continued in 2024, leading to some inspiring interactions and relationships between young clinicians and rare disease patient communities, with students attending patient group annual meetings, and at least one example of a student joining a medical advisory board.

## Lasting impact

The Student Voice Prize has also been an excellent opportunity to engage early-career healthcare professionals in the art of contributing to medical literature. There is a paucity of peer-reviewed medical literature that truly reflects either the lived patient experience of rare disease, or the unique perspective of early career healthcare professionals. The annual publication of the winning essay is a great incentive for students to take part and even for those who don’t win, this opportunity is valuable experience for future attempts at publishing literature. Winning Student Voice Prize essays have made a significant contribution to our understanding of the rare disease lived experience. Rebecca Nunn’s essay, ‘“It’s not all in my head!” – The complex relationship between rare diseases and mental health problems’, has 40 citations and the winning essays have a combined total of over 46,000 article views to date [1]. The interest in the lived experience and healthcare student perspective cannot be understated. Furthermore, the competition has launched long-term careers in rare disease with a number of participants going on to become involved in PAGs, take part in The Global Genes Rare Compassion Project, become Medics for Rare Disease Ambassadors or pursue careers in rare disease specialisms. Shaping a clinical community to have an understanding of rare disease is central to the mission of Medics for Rare Disease, and essential for improving outcomes for those living with rare conditions in years to come.

## Appealing to student HCPs

As the competition evolved we became more knowledgeable about which topics and question types appealed most to student participants. The case study question has always been the most popular and the winner of this question is the person who demonstrates the deepest self-reflection based on the lived experience. This shows that the students want to have a real appreciation of what life is like when you live with a rare condition. Another popular approach to the essays is through comparison - looking at experiences across different countries, demographics or prevalence. This demonstrates a curiosity amongst student authors about why some people face greater challenges and health inequities than others based on circumstances rather than disease pathophysiology.

As the years progressed we took the opportunity to create questions that appealed more to our audience of student healthcare professionals; themes included: mental health, inequality, social challenges, and their overlap with rare diseases and healthcare in general. This approach has not only helped to grow the competition but gather more unique perspectives from the world’s future healthcare professionals and - perspectives which may not otherwise be readily heard.

## Rare lives - equal rights?

Participants of the Student Voice Prize can always be relied on to highlight the inequities of care and opportunity experienced by those living with rare disease. The concept of social justice - the fair and equitable treatment of all individuals and groups within society - has been long understood and discussed (if not achieved), especially when thinking about marginalised groups. However we rarely hear the use of this term used in relation to the large (350 million) yet marginalised global population of people living with a rare disease.

The Student Voice Prize essays now contribute a major portion of articles focusing on social justice and rare disease. And that’s just the published articles - we find ourselves wishing we could cite the essays that were not published by OJRD and yet are so valuable in their insights. The competition has become an area of advocacy we know will reliably highlight the injustices in how the rare disease community is catered for in society - all in the words of student healthcare professionals.

## The student voice

Year after year, participants in the The Student Voice Prize have been highlighting the social injustice experienced by the rare community. They come with fresh eyes, ready to listen and learn from lived experience, and are shocked by how the rare community is treated unfairly in healthcare and society. Whether it’s two sisters unable to receive treatment for their rare inflammatory condition that is approved but not funded for their rare indication, or Sarah’s diagnosis of Marfan Syndrome, only after her two siblings had died of complications [2, 3]. In a 2021 essay, one Runner Up quotes her interviewee who was told *“What is Leigh Syndrome? If you had MS*,* I could help you”* by their neurologist [4]. It is the social injustice that they see as important to highlight - it’s the avoidable suffering that they want to shout about. Another Runner Up expertly highlighted the predictable and preventable mental health deterioration of a carer who is demoralised due to a staggering lack of support for her son [5].

We are glad to see that in 2025 one theme was to take up space in rare disease advocacy more than any other: social justice for the rare disease community. The rare advocacy community has finally turned its focus to what the students have been pointing out for years: low prevalence is not an excuse for suboptimal care and support. Metabolic Support UK’s Living Well campaign focuses on the need to educate and support people living with a rare disease to understand their rights, and to encourage policy to support people to “live well” with a rare condition, rather than only focus on medical intervention. The Rare Barometer survey on societal inclusion showed that only 21% of applicable respondents were fully participating in education and the unemployment rate is higher in people living with rare disease than the average rate in the European Union (23% vs. 6.1%) [6]. And of course the Lancet Commission on Rare Diseases with its focus on whether human rights are being observed for the rare community. We are all born equal in dignity and rights and we are proud that, through OJRD, The Student Voice Prize has contributed to our understanding of barriers and opportunities for achieving social justice for those impacted by rare disease.

## The next decade

We set out to educate HCP students and they gave back more than we could ever have anticipated. We commend Orphanet Journal of Rare Diseases and Springer Nature Journals for their commitment to publishing student reflections and insights. Long may this collaboration continue.
